# Comparison of Prognostic Gene Profiles Using qRT-PCR in Paraffin Samples: A Retrospective Study in Patients with Early Breast Cancer

**DOI:** 10.1371/journal.pone.0005911

**Published:** 2009-06-15

**Authors:** Enrique Espinosa, Iker Sánchez-Navarro, Angelo Gámez-Pozo, Álvaro Pinto Marin, David Hardisson, Rosario Madero, Andrés Redondo, Pilar Zamora, Belén San José Valiente, Marta Mendiola, Manuel González Barón, Juan Ángel Fresno Vara

**Affiliations:** 1 Service of Oncology, Hospital La Paz, Madrid, Spain; 2 Service of Pathology, Hospital La Paz, Madrid, Spain; 3 Service of Statistics, Hospital La Paz, Madrid, Spain; Baylor College of Medicine, United States of America

## Abstract

**Introduction:**

Gene profiling may improve prognostic accuracy in patients with early breast cancer, but this technology is not widely available. We used commercial assays for qRT-PCR to assess the performance of the gene profiles included in the 70-Gene Signature, the Recurrence Score and the Two-Gene Ratio.

**Methods:**

153 patients with early breast cancer and a minimum follow-up of 5 years were included. All tumours were positive for hormonal receptors and 38% had positive lymph nodes; 64% of patients received adjuvant chemotherapy. RNA was extracted from formalin-fixed paraffin-embedded (FFPE) specimens using a specific kit. qRT-PCR amplifications were performed with TaqMan Gene Expression Assays products. We applied the three gene-expression-based models to our patient cohort to compare the predictions derived from these gene sets.

**Results:**

After a median follow-up of 91 months, 22% of patients relapsed. The distant metastasis-free survival (DMFS) at 5 years was calculated for each profile. For the 70-Gene Signature, DMFS was 95% -good prognosis- versus 66% -poor prognosis. In the case of the Recurrence Score, DMFS was 98%, 81% and 69% for low, intermediate and high-risk groups, respectively. Finally, for the Two-Gene Ratio, DMFS was 86% versus 70%. The 70-Gene Signature and the Recurrence Score were highly informative in identifying patients with distant metastasis, even in multivariate analysis.

**Conclusion:**

Commercially available assays for qRT-PCR can be used to assess the prognostic utility of previously published gene expression profiles in FFPE material from patients with early breast cancer. Our results, with the use of a different platform and with different material, confirm the robustness of the 70-Gene Signature and represent an independent test for the Recurrence Score, using different primer/probe sets.

## Introduction

A key area in the management of women with early breast cancer is the selection of adjuvant therapy, which depends on the use of prognostic and predictive factors. Chemotherapy is usually recommended in the presence of adverse factors, such as positive lymph nodes, size>1 cm, histological grade>1 or negative hormonal receptors. These factors are included in guidelines or specific software to help decision making [1], [2], but using these criteria leads to unnecessary treatment in many women, either because they would not relapse in the absence of adjuvant chemotherapy or because they would suffer a relapse anyway [3].

Gene expression profiles may improve prognostic and predictive information in breast cancer patients. Two of these profiles, the 70-Gene Signature (MammaPrint™) and the Recurrence Score (OncoType DX™) are being evaluated in phase III studies, with randomization based on the results of the assays [4], [5]. The 70-Gene Signature is suitable for patients with either ER-positive or ER-negative tumours, whereas the Recurrence Score has to be used in ER-positive tumours. Another group has reported a Two-Gene Ratio (HOXB13/IL17BR) predicting disease-free survival in patients with early-stage, ER-positive breast cancer who received adjuvant tamoxifen [6]. An RT-PCR based method to assess this ratio from paraffin-embedded tissue samples is now commercially available (Theros H/I; bioTheranostics).

Some centres already use gene profiles to identify patients with very low risk of relapse who may not need adjuvant chemotherapy. The major restraints for the widespread application of such tests are some reservations regarding their cost/effectiveness ratio and the lack of availability, because samples must be sent to central laboratories for processing. The need for fresh-frozen (FF) material in some cases adds to these restraints, as the process of collecting, processing and storing FF samples for large scale studies is difficult. However, the use of gene profiles would be facilitated if centralized processing was not required and formalin-fixed paraffin-embedded (FFPE) samples could be used. FFPE samples are stable at room temperature, easily storable and, most importantly, they constitute a widely available archive of clinical samples linked to clinical information.

In the present study, we assessed the performance of the above referred gene expression profiles by using commercially available assays for qRT-PCR in FFPE samples.

## Results

One hundred fifty-three patients diagnosed between February-95 and March-03 were included. Table 1 shows their clinical features. Median age was 58 years and median follow-up was 91 months for the whole group. Sixty-six patients (43%) had a mastectomy, whereas the remaining underwent a conservative surgery followed by adjuvant radiation. All patients received adjuvant tamoxifen for five years and 97 (63%) underwent adjuvant chemotherapy. Thirty-four patients (22%) had a distant relapse, of which 17 died and 7 were lost for follow-up after the relapse. Among 119 patients without distant relapse, four had a local/regional recurrence successfully treated with surgery.

**Table 1:** Clinical characteristics of the patients.

	Number of patients (percentage)
Age	Median 58, range: 29–82
T	
1	77 (50.3%)
2	76 (49.7%)
N	
0	96 (62.7%)
1	57 (37.3%)
Stage	
I	61 (39.9%)
IIa	51 (33.3%)
IIb	41 (26.8%)
Hormone receptors	
ER+/PgR−	30 (19.6%)
ER+/PgR+	110 (71.9%)
ER+/PgR unknown	12 (7.9%)
ER−/PgR+	1 (0.7%)
Grade	
1	29 (18.9%)
2	64 (41.8%)
3	59 (38.6%)
x	1 (0.7%)
Chemotherapy	
No chemotherapy	56 (36.6%)
CMF	42 (27.4%)
Anthracycline-based	55 (35.9%)

All tumours were positive for oestrogen and/or progesterone receptors. All patients had received adjuvant tamoxifen.

**Figure 1:**
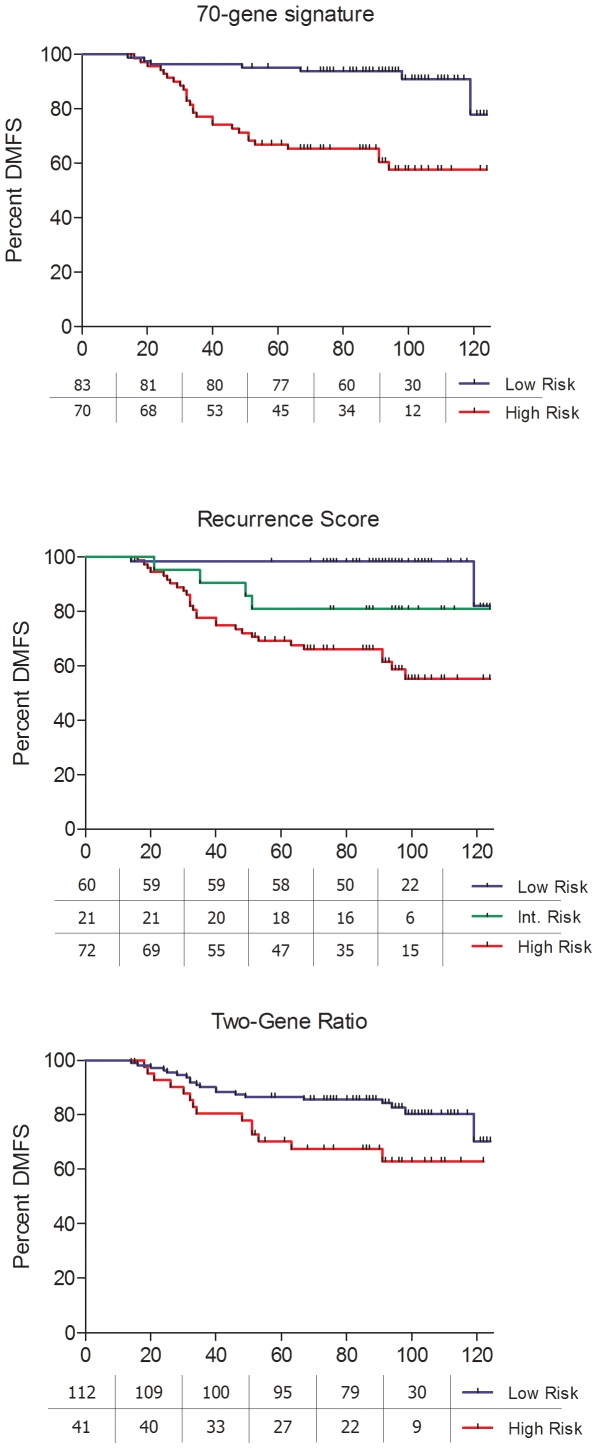
Kaplan-Meier plots for distant metastasis-free survival for the whole group.

**Figure 2:**
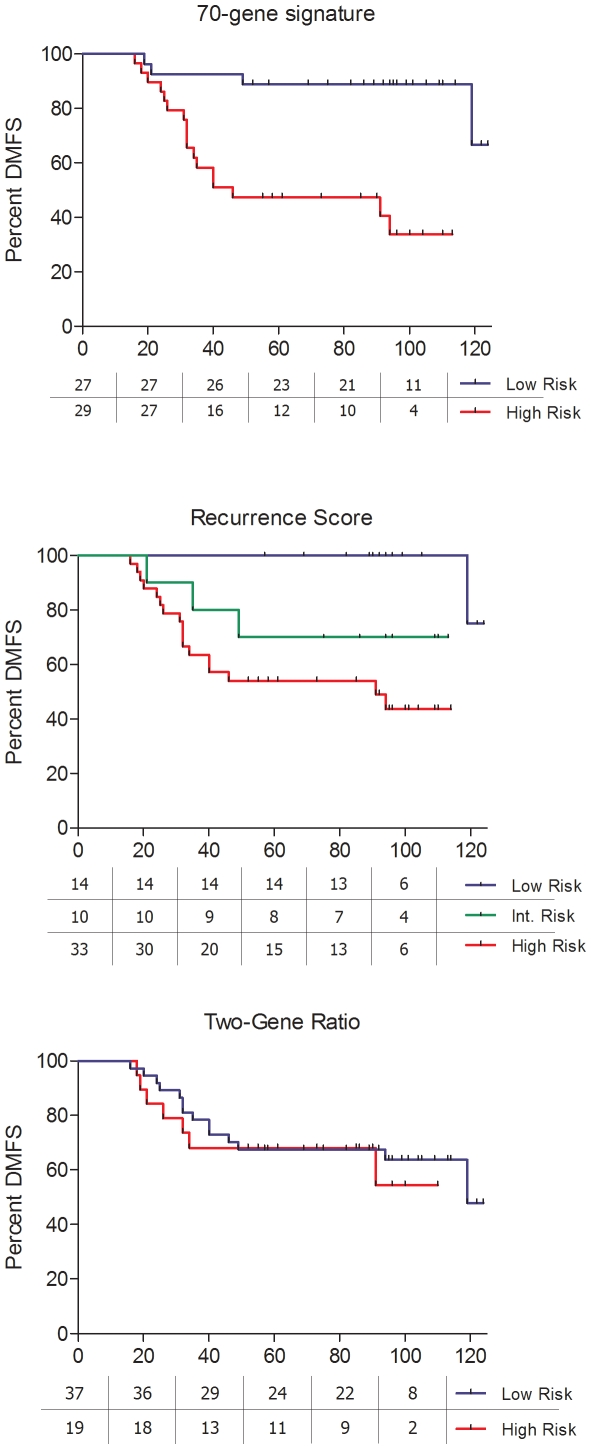
Survival analysis in patients who did not receive chemotherapy.

qRT-PCR reactions worked correctly for all genes included in the Recurrence Score, both genes of the Two-Gene Ratio, and all 60 we could include from the 70-Gene Signature. Raw data appear in supplementary Table S1. In the univariate analysis, lymph-node status, tumour grade, size and the three gene profiles were significant predictors of DMFS (lymph-node status p = 0.001; tumour grade p<0.001; size p = 0.002; 70-Gene Signature p<0.001; Recurrence Score<0.001; Two-gene Ratio p = 0.023). DMFS at five years for every profile was as follows: for the 70-Gene Signature, 95% good prognosis versus 66% poor prognosis; for the Recurrence Score profile, 98% low-risk versus 81% intermediate risk versus 69% high-risk; and in the case of the Two-Gene Ratio, 86% favourable versus 70% unfavourable (figure 1). We restricted the univariate analysis to patients who did not receive adjuvant chemotherapy and the three profiles still found significant differences (figure 2).

Cramer's V statistic was performed in the profile-to-profile comparison and, for this purpose, we combined the low and intermediate Recurrence Score categories. The concordance of the Two-Gene Ratio with either the 70-Gene Signature or the Recurrence Score was 0.3 in both cases. Concordance was 0.6 between the 70-Gene Signature and the Recurrence Score, indicating a strong correlation. Most tumours classified as having a low risk of recurrence by one the three models were classified as such by the other two, although the low-risk group defined by the Two-Gene Ratio was poorly predicted by the 70-Gene Signature and the Recurrence Score. When comparing high risk tumours, again there was a good correlation between the 70-Gene Signature and the Recurrence Score, but rather poor for the Two-Gene Ratio. Table 2 shows the intersection between profiles.

**Table 2:** Concordance among the three profiles.

Good prognosis by…	Coincidence cases	Coincidence cases
70-Gene Signature	Recurrence Score	Two-Gene Ratio
n = 83; 54%	n = 66; 79%	n = 71; 85%
Recurrence Score	70-Gene Signature	Two-Gene Ratio
n = 81; 53%	n = 66; 81%	n = 68; 84%
Two-Gene Ratio	70-Gene Signature	Recurrence Score
n = 112; 73%	n = 71; 63%	n = 68; 61%
Poor prognosis by…	Coincidence cases	Coincidence cases
70-Gene Signature	Recurrence Score	Two-Gene Ratio
n = 70; 46%	n = 55; 78%	n = 29; 41%
Recurrence Score	70-Gene Signature	Two-Gene Ratio
n = 72; 47%	n = 55: 76%	n = 28; 39%
Two-Gene Ratio	70-Gene Signature	Recurrence Score
n = 41; 27%	n = 29; 71%	n = 28; 68%

To assess the discrimination capability of each prognostic profile at 5 years, Harrell's bias corrected concordance index was calculated. The values were: Recurrence Score = 0.73>70-Gene Signature = 0.70>Two-Gene Ratio = 0.59.

We also performed a multivariate Cox proportional hazards analysis to evaluate the prognostic value of each gene-expression based model individually. The analysis also included tumour size, nodal status and tumour grade. The 70-Gene Signature and the Recurrence Score were significant predictors of DMFS (Table S2, supporting information), indicating that those gene expression profiles added important prognostic information beyond that provided by clinical factors. The Two-Gene Ratio was not a significant predictor in the multivariate analysis. Node status remained the only clinical factor with significant value in all cases. Histological grade was significant for the cases of Recurrence Score (p = 0.043) and Two-Gene Ratio (p = 0.002).

**Figure 3:**
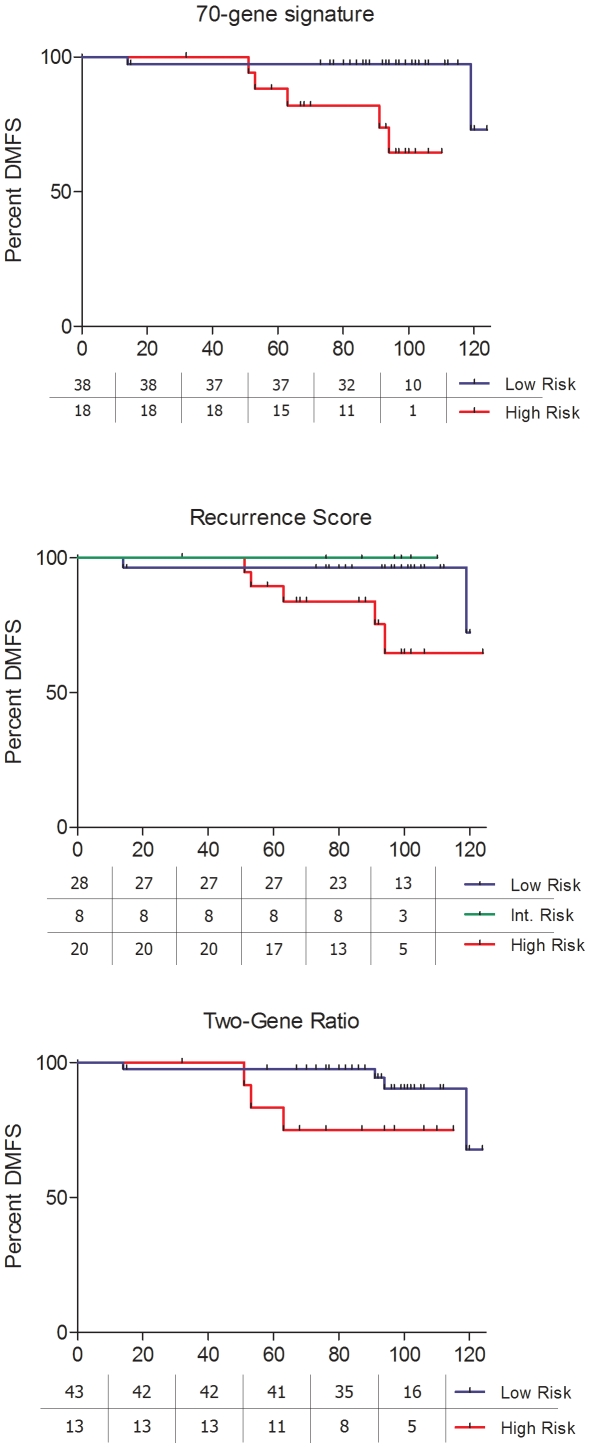
Survival analysis in patients with positive lymph nodes.

Likewise, the 70-Gene Signature and the Recurrence Score detected striking differences for the subgroup of patients with positive lymph nodes, whereas differences were not significant in the case of the Two-Gene Ratio (figure 3).

Adjuvant! Online estimated that chemotherapy would reduce the rate of relapse by less than 5% in 37% of patients. A threshold of 5% was selected because it is used by many clinicians to discourage the use of chemotherapy. By contrast, the three gene profiles allocated more patients in low-risk groups: 46% the 70-Gene Signature, 53% the Recurrence Score (low + intermediate risk) and 73% the Two-Gene Ratio. The multivariate analysis including Adjuvant! Online showed that both the 70-Gene Signature and the Recurrence Score remained significant (p<0.005), while the Two-Gene Ratio was not (p = 0.078) (Table 3). In our population, the Nottingham Prognosis Index did not show significant value to define prognosis in the univariate analysis (p = 0.061), so that it was not included in the multivariate analysis.

**Table 3:** Multivariate analysis comparing Adjuvant! Online with gene expression classifiers.

	Adjuvant! Online		
	HR	CI 95%	p value
Adjuvant! Online	10.814	2.589–45.173	0.001
70-Gene Signature	4.218	1.800–9.883	0.001
Adjuvant! Online	7.458	1.764–31.533	0.006
Recurrence Score			0.003
(Interm. risk vs. Low risk)	4.132	0.745–22.906	0.105
(High risk vs. Low risk)	10.098	2.343–43.518	0.002
Adjuvant! Online	6.451	1.529–27.226	0.011
Two-Gene Ratio	1.864	0.932–3.727	0.078
Adjuvant! Online	10.051	2.400–42.101	0.002

The gain in predictive accuracy from adding each of the analyzed signatures to the Nottingham Prognostic Index and Adjuvant! Online clinical systems is presented in Table 4. The individual performance for DMFS of Nottingham Prognostic Index and Adjuvant! Online was comparable (0.239 and 0.236, respectively). The performance of the 70-Gene Signature (0.234) was in line with that of both clinical staging systems, whereas the Recurrence Score showed the best performance (0.225). The addition of the 70-Gene Signature and the Recurrence Score to any of the clinical staging systems decreased their predictive inaccuracy. The gain by adding the 70-Gene Signature in explained variation was 9.2%, and over 10% for the Recurrence Score in this dataset. The Two-Gene Ratio did not improve the clinical staging systems explained variation.

**Table 4:** Explained variation and predictive inaccuracy for DMFS.

	Predicitve inaccuracy	Explained variation (%)
No Predictor	0.275	
NPI	0.239±0.006	13.3±1.0
Adjuvant!	0.236±0.002	13.9±0.9
70-Gene S.	0.234±0.003	14.8±1.0
RS	0.225±0.003	18.1±0.9
Two-Gene Ratio	0.264±0.002	3.4±0.9
NPI & 70-Gene S.	0.213±0.004	22.4±1.3
Adjuvant! & 70-Gene S.	0.211±0.003	23.1±1.2
NPI & RS	0.210±0.005	23.7±1.5
Adjuvant! & RS	0.204±0.004	25.8±1.4
NPI & Two-Gene Ratio	0.237±0.006	13.8±1.5
Adjuvant! & Two-Gene Ratio	0.232±0.004	15.5±1.5
Gain by adding 70-Gene S. to NPI	0.025	9.2
Gain by adding 70-Gene S. to Adjuvant!	0.025	9.2
Gain by adding RS to NPI	0.028	10.4
Gain by adding RS to Adjuvant!	0.033	11.8
Gain by adding Two-Gene Ratio to NPI	0.001	0.5
Gain by adding Two-Gene Ratio to Adjuvant!	0.004	1.5

NPI: Nottingham Prognostic Index, RS: Recurrence Score, 70-Gene S.: 70-Gene Signature. Data presented as the mean±standard error.

## Discussion

Gene profiles help to determine prognosis in patients with early breast cancer and some of them are being used to make clinical decisions. If gene profiles become part of the standard pathological workup in early breast cancer in the future, general availability will be required and the use of FFPE material would be very useful. Several studies have demonstrated the feasibility of using FFPE tissues to perform gene expression profiling by qRT-PCR. Although RNA degradation leads to a loss of amplifiable templates, optimized normalization strategies could effectively compensate for this bias [4], [7], [8].

We compared the performance of three gene profiles -70-Gene Signature, Recurrence Score and Two-Gene Ratio- in patients with early breast cancer. The material was FFPE tissue and the technique qRT-PCR in all cases, in an attempt to simplify technical procedures. The 70-Gene Signature was initially described in young women who had not received adjuvant therapy, but further research has confirmed its validity in other groups [5], [9], [10], [11], and its applicability in patients with either positive or negative hormonal receptors [12]. On the other hand, the Recurrence Score is aimed at tumours expressing hormonal receptors and provides prognostic and predictive information in patients treated with tamoxifen [4], [13], [14]. The Two-Gene Ratio was developed and validated in early-stage hormonal receptor positive patients that had received adjuvant tamoxifen therapy [6], [15]. So the populations where each of these profiles was initially described vary according to their clinical, pathological and therapeutic features, thus hindering any direct comparison. In our study, the three profiles identified groups of patients with significant differences in DMFS, although only the 70-Gene Signature and the Recurrence Score offered significant value in the multivariate analysis, showing a high correlation between them. The other profile -Two-Gene Ratio- has recently been improved by incorporating five genes related to tumour grade [16]. This five-gene set was comparable to a more complex 97-gene genomic grade index in multiple data sets [17].

Clinical comparisons have not been performed among profiles, although one study by Fan et al. evaluated five gene sets in a data set of patients [18]. This study demonstrated significant agreement among four out of the five profiles, namely, intrinsic subtypes, 70-Gene Signature, Recurrence Score and Wound-Signature. The conclusion was that they probably track a common set of biologic phenotypes. In agreement with the study by Fan et al, we find a high concordance between the 70-Gene Signature and the Recurrence Score and a lack of reproducibility of the Two-Gene Ratio. However, there are important differences between both studies. Firstly, we hereby present an independent set of patients who received more aggressive therapy than those included in the other study; this could have improved outcome in our patients. Secondly, all of our samples derived from FFPE tissue. Thirdly, the technique we used was qRT-PCR for the three profiles, whereas Fan et al used the original assay for the 70-Gene Signature and translated the PCR values of the Recurrence Score into a microarray dataset. In other words, we used a different platform for the 70-Gene Signature and different, commercial available probes for the Recurrence Score. To our knowledge, our study is the first fully independent application of the Recurrence Score algorithm, because other investigators sent samples to a central laboratory for processing [19].

Fan et al. showed that microarray data would be applicable to assess the Recurrence Score and the Two-Gene Ratio (originally developed with PCR), so the opposite assumption may be also correct. On the other hand, we previously demonstrated that qRT-PCR can be used to assess the 70-Gene Signature profile [20]. Likewise, the present study proves that the 70-Gene Signature can also be determined from FFPE material. This work solves a technical difference that gave certain advantage to the Recurrence Score over the 70-Gene Signature in their attempt to burst in the clinic, i.e., the use of FFPE material. Our results with the use of a different platform and with different material confirm the robustness of the 70-Gene Signature and the Recurrence Score. In other words, the value of these profiles cannot be attributed to the use of a specific platform, but to the algorithms and the genes themselves. Our results are in agreement with those of other investigators seeing that previously reported prognostic signatures, despite differences in gene lists, carry similar prognostic information [21], [22].

In our series, gene profiles outperformed clinical data to identify patients with low risk of relapse. The use of Adjuvant! Online would have led to recommend chemotherapy in 63% of patients whereas few patients lied in high-risk groups determined by gene expression profiles. A recent study with hormone receptor-positive breast cancer and zero to three positive axillary nodes also demonstrated that the Recurrence Score is a more accurate predictor of relapse than standard features included in Adjuvant! Online [23].This is important considering that our series consisted of patients with small tumours and none or up to three positive lymph nodes, i.e., good prognosis tumours. Differences could have been bigger in a non-selected population including tumours with unfavourable clinical features, as shown in other studies [13], [24]. Although subgroup analysis must be viewed with caution, we also found that in patients with positive lymph nodes, a favourable 70-Gene Signature or a low-risk Recurrence Score was still associated with an excellent prognosis. Similar results were seen in the group not receiving chemotherapy.

Additionally, we showed that the Recurrence Score and the 70-Gene Signature improved the predictive accuracy of commonly used clinical systems, whereas the Two-Gene Ratio did not. This is relevant because experts in the field agree that these new techniques should provide more accurate information than classical factors to be incorporated into clinical practice. Moreover, these gene expression classifiers should not be regarded as a tool to replace standard pathological and clinical criteria, but should instead be integrated with clinical parameters [25].

Phase III trials have been initiated to determine whether two of them –the 70-Gene Signature and the Recurrence Score- may reduce the need for adjuvant chemotherapy in patients with low risk of relapse. Of course, this does not support the indiscriminate use of this new technology in the clinic, at least by present standards. But if phase III trials demonstrate the validity of either the 70-Gene Signature or the Recurrence Score, a substantial number of patients could be treated without adjuvant chemotherapy in the future. Patients with N0 disease would benefit first from this strategy, as many of them (particularly young women) are offered chemotherapy if there is an adverse factor, such as high grade or size >2 cm. There is also a possibility that some patients with positive lymph nodes could do so in the future. The field is evolving very rapidly and new contributions will be needed to improve the accuracy of the information provided by currently available profiles.

In summary, we verified the performance of some gene profiles, particularly the 70-Gene Signature and the Recurrence Score by using qRT-PCR in FFPE samples with commercially available assays. Our study opens the possibility to simplify the procedures to perform high-throughput techniques in the general population of patients with breast carcinoma. However, before this technology is taken to the clinic, ongoing phase III trials should validate the utility of prognostic gene profiles.

## Materials and Methods

### Ethics statement

At the time of initial diagnosis, all patients had provided consent in the sense that their tumour samples could be used for investigational purposes. Institutional approval from our ethical committee was obtained for the conduct of the study (Comité Ético de Investigación Clínica). Data were analyzed anonymously. Patients provided written consent so that their samples and clinical data could be used for investigational purposes.

### Patients and clinical data

The study population consisted of women with early breast cancer. Inclusion criteria were: invasive ductal carcinoma, stage I or II (TNM classification, 2002), positive for oestrogen and/or progesterone receptors and appropriate therapy. Appropriate therapy should include either mastectomy or tumorectomy plus adjuvant radiotherapy, adjuvant hormonal therapy for 5 years in all patients, and adjuvant anthracycline-based chemotherapy in N+ or in N0 patients with poor prognostic features. A minimum follow-up of 5 years was also required. The following data were recorded and tabulated: age at diagnosis, size of primary tumour, lymph node stage, number of positive nodes, grade of differentiation, hormonal receptors, adjuvant therapy (either radiation, hormones or chemotherapy), date of relapse or last follow-up, site of relapse, cause of death.

### RNA Isolation and cDNA Synthesis

An experienced pathologist evaluated H&E preparations to select samples with at least 70% of tumour cells. Fifteen sections 5 µm each from every FFPE sample were deparaffinized with xylene and washed with ethanol in decreasing concentrations (100%, 90% and 70%). RNA was then extracted with the Master Pure™ Kit (Epicentre).

Isolated total RNA was quantified and qualitatively assessed using spectrophotometer OD_260_ measurements and agarose gel electrophoresis. We normalized to total RNA input; therefore, first-strand cDNA was synthesized from 1 µg of total RNA, using random primers, according to the High Capacity cDNA Reverse Transcription Kit protocol (Applied Biosystems). The complete reaction mixes were incubated at 25°C for 10 min and 37°C for 120 min.

### Quantitative RT-PCR

qRT-PCR amplifications were performed with TaqMan Gene Expression Assays products in an ABI PRISM 7900 HT Sequence Detection System (Applied Biosystems). The reactions were carried out using the TaqMan Low Density Arrays (TLDAs, Applied Biosystems) containing 50 µL TaqMan Universal PCR Master Mix (Applied Biosystems) and 50 µL of a cDNA template corresponding to 100 ng total RNA per channel of the microfluidic card.

### Gene selection

We configured a TLDA series to analyze those genes included in the 70-Gene Signature [5], the Recurrence Score [4] and the Two-Gene Ratio [6] with TaqMan Gene Expression Assays available. Table S1 (supporting information) shows the assays we used to study the genes included in each profile as well as the housekeeping genes.

Although RNA degradation in FFPE samples leads to a loss of amplifiable templates, optimized normalization strategies can effectively compensate for this bias [7], [8]. We have generated a normalization model called NorMean (unpublished data). This model ranks housekeeping genes according to their capacity to control for several levels of experimental variability in qRT-PCR. Using this ranking, we calculated different normalization factors by stepwise inclusion of control genes and geometric averaging of their expression levels. The optimal number of control genes for normalization was determined by comparing the percentage of significantly correlated genes between FF and FFPE materials using each normalization factor.

### Calculations for gene expression profiles

Average cycling threshold (Ct) values, defined as the point at which the fluorescence rises above the background fluorescence [26], were obtained using the SDS 2.2 software (Applied Biosystems). The maximum Ct value was set at 40. These Ct values were recorded in Microsoft Excel 2003 for subsequent calculations. The methods originally described for the 70-Gene Signature [10], the Recurrence Score [4] and the Two-Gene Ratio [6], [15] were used to analyze the performance of these profiles.

#### 70-Gene Signature

Sixty out of the 70 genes were included [10], as the remaining 10 were not available for TLDAs by the time the experiment was performed. One previous study demonstrated that a reduction of the signature had little impact on the performance of the classifier [12]. Relative expression level of each target gene was expressed as ΔCt = Ct_ref_−Ct_target_. Normalization was performed using the geometric mean of the best housekeeping gene set (IPO8, POLR2A, UBC and SDHA). As the 70-gene signature was defined using microarrays, normalized gene Cts values were z-score transformed. The mean good-prognosis profile was calculated averaging the gene expression values of each gene for the patients without recurrence. Pearson's correlation coefficient between this mean good-prognosis profile and each patient's gene expression profile was calculated. A value threshold = 0 was used, as previously described [12]. Although the validity of calculating correlation coefficients in this way is questionable [27], we decided to use it to resemble as much as possible the methodology described by van't Veer et al [5] and others [12].

#### Recurrence Score

Briefly, expression of each gene was normalized relative to the expression of the reference genes (ACTB, GAPDH, GUS, RPLP0 and TFRC). Reference-normalized expression measurements were calculated as described by Paik et al., so that one unit increase in reference-normalized expression measurements reflects approximately a 2-fold increase in RNA. We substituted GSTM1 by GSTM3 because no GSTM1 probe was available for the TLDAs, as described by the authors in the patent information (Baker J et al, http://www.freepatentsonline.com/20050048542.html)

The RS algorithm was then used to generate an unscaled Recurrence Score for each patient. Values were scaled and patients were assigned to the low, intermediate or high-risk group using the RS cut-offs previously described [4].

#### Two-Gene Ratio

Relative expression levels of HOXB13 and IL17BR were expressed as ΔCt = Ct_ref_−Ct_target_. Cts for the four reference genes (ACTB, HMBS, SDHA and UBC) were averaged to obtain Ct_ref_. These values were z-transformed for each gene and the Two-Gene Ratio was calculated taking the difference and using a cut off point of 1.0, as described [15].

### Statistical Analysis

The prognostic value of each gene-expression–based model was evaluated by log-rank test. We also applied multivariate Cox proportional-hazards analysis to each profile individually in a model including tumour grade (2 vs. 1 and 3 vs. 1), size (>2 cm vs. ≤2 cm) and nodal status (one to three positive nodes vs. no positive nodes). We also applied multivariate Cox analyses to each profile in a model including all clinical parameters that resulted significant in the univariate analysis. Distant metastasis-free survival (DMFS) was the end point, similarly to other studies of gene profiles in breast cancer.

Two-way contingency-table analyses and the calculation of Cramer's V statistic were also performed to measure the strength of the association between the different profiles [18]. As both the 70-Gene Signature and the Two-Gene Ratio establish two groups with high and low risk, this analysis required that the low and intermediate groups of the Recurrence Score were combined.

To assess model accuracy (discrimination) at five years, Harrell's bias corrected concordance index was calculated. Models were refit 500 times with the bootstrap resampling technique. The concordance index is the percentage of patient pairs in which the predicted and observed outcomes are in agreement; i.e., the probability that for two patients chosen at random, the patient who had the event first had a higher probability of having the event according to the model. c = 0.50 represents agreement by chance; c = 1.0 represents perfect discrimination [28]. Concordance is essentially the Wilcoxon-Mann-Whitney statistic for comparing predictions in the two outcome groups, and it is identical to the area under a receiver operating characteristic curve (ROC) [29].

The gain in predictive accuracy of each classifier, as compared with common clinical staging systems, was investigated using the method of Schemper and Henderson, implemented in the R package software as previously described [21], [25], [30], [31].

All statistical analyses were performed with the SPSS v9.1 software package, GraphPad Prism v5.00 and “R” v 2.2 with the Design software package v2.0-12. All *P* values were two-sided, and *P*<0.05 was considered statistically significant.

## Supporting Information

Table S1 Assays and raw data.
(0.34 MB XLS)

Table S2 Multivariate analysis with individual clinical factors
(0.05 MB DOC)
