# Supplementary material for: Comparison of Prognostic Gene Profiles Using qRT-PCR in Paraffin Samples: A Retrospective Study in Patients with Early Breast Cancer
Source: PLoS One. 2009 Jun 15;4(6):e5911. doi: 10.1371/journal.pone.0005911 (PMC2698956; doi:10.1371/journal.pone.0005911)
Supplement: Table S2 — Multivariate analysis with individual clinical factors (0.05 MB DOC) [file pone.0005911.s002.doc]

**Supplementary table II.** Multivariate análisis with individual clinical factors.

|  | **70-Gene Signature** | | |
| --- | --- | --- | --- |
|  | HR | CI 95% | p value |
| Tumour size (>2cm vs. <2cm) | 1,398 | 0.571 - 3.424 | 0.463 |
| Node (1-3 vs. 0) | 2.706 | 1.303 - 5.622 | **0.008** |
| Grade  (2 vs.1)  (3 vs. 1) | 0.819  2.349 | 0.160 - 4.181  0.473 - 11.672 | 0.113  0.810  0.297 |
| 70-Gene Index | 3.506 | 1.331 - 9.229 | **0.011** |
|  | **Recurrence Score** | | |
|  | HR | CI 95% | p value |
| Tumour size (>2cm vs. <2cm) | 1.172 | 0.487 - 2.822 | 0.724 |
| Node (1-3 vs. 0) | 2.497 | 1.207 - 5.168 | **0.014** |
| Grade  (2 vs.1)  (3 vs. 1) | 0.682  2.267 | 0.135 – 3.453  0.494 – 10.404 | **0.043**  0.644  0.292 |
| Recurrence Score  (Low risk vs. Interm. risk)  (Low risk vs. High risk) | 4.466  8.180 | 0.787 – 25.331  1.767 – 37.861 | **0.021**  0.091  **0.007** |
|  | **Two-Gene Ratio** | | |
|  | HR | CI 95% | p value |
| Tumour size (>2cm vs. <2cm) | 1.597 | 0.674 – 3.782 | 0.287 |
| Node (1-3 vs. 0) | 2.314 | 1.132 – 4.729 | **0.021** |
| Grade  (2 vs.1)  (3 vs. 1) | 1.076  4.932 | 0.216 - 5.367  1.111 - 21.903 | **0.002**  0.929  **0.036** |
| Two-Gene Ratio | 1.181 | 0.579 - 2.410 | 0.647 |
|  | **Classical Factors** | | |
|  | HR | CI 95% | p value |
| Tumor size (>2cm vs. <2cm) | 1.613 | 0.683 - 3.811 | 0.276 |
| Node (1-3 vs. 0) | 2.334 | 1.145 - 4.757 | **0.020** |
| Grade  (2 vs.1)  (3 vs. 1) | 1.074  5.139 | 0.215 - 5.359  1.168 - 22.612 | **0.001**  0.931  **0.030** |
